# Bridging the Gap in Pain Measurement with a Brain-Based Index

**DOI:** 10.3390/ijerph23010033

**Published:** 2025-12-24

**Authors:** Colince Meli Segning, Abderaouf Bouhali, Luis Vicente Franco de Oliveira, Claudia Santos Oliveira, Rubens A. da Silva, Karen Barros Parron Fernandes, Suzy Ngomo

**Affiliations:** 1 Laboratoire de Recherche Biomécanique et Neurophysiologique en Réadaptation Neuro-Musculo-Squelettique (*Lab BioNR*), Centre Intersectoriel en Santé Durable (CISD), Université du Québec à Chicoutimi (UQAC), Saguenay, QC G7H 2B1, Canada; cmsegnin@uqac.ca (C.M.S.); abouhali1@etu.uqac.ca (A.B.); rubens_dasilva@uqac.ca (R.A.d.S.); karen.parron@pucpr.br (K.B.P.F.); 2 Post-Graduation Program in Human Movement and Rehabilitation, Centro Universitário UniEvangélica, Anápolis 75083–515, GO, Brazil; luis.oliveira@unievangelica.edu.br (L.V.F.d.O.); claudia.oliveira@unievangelica.edu.br (C.S.O.); 3 Centre Intégré de Santé et Services Sociaux du Saguenay-Lac-Saint-Jean (CIUSSS SLSJ), Specialized Geriatrics Services-Hôpital de La Baie, Saguenay, QC G7H 7K9, Canada; 4 Graduate Program in Health Sciences (PPGCS), School of Medicine, Pontifical Catholic University of Parana (PUCPR), Curitiba 80215-901, PR, Brazil; 5 School of Medicine, Pontifical Catholic University of Parana (PUCPR), Londrina 86072-000, PR, Brazil

**Keywords:** electroencephalography (EEG), beta frequency band, Hilbert transform, pain identification and quantification (Piq), longitudinal design, cross-sectional design, proposed graded scale for Piq_β_

## Abstract

**Highlights:**

**Public health relevance—How does this work relate to a public health issue?**

- Pain intensity assessment relies mainly on self-report or clinician-based reporting, which limits accuracy in several clinical contexts.
- Piq_β_ is introduced as an EEG-based index derived from beta-band cortical activity that objectively identifies and quantifies pain.

**Public health significance—Why is this work of significance to public health?**

- Chronic pain is a major global burden that contributes to polypharmacy, addiction, and reduced social participation.
- Piq_β_ may help strengthen the physiological validation of pain intensity, support recognition of the patient’s pain experience, and offer an additional element for rehabilitation strategies.

**Public health implications—What are the key implications or messages for practi-tioners, policy makers and/or researchers in public health?**

- Piq_β_ can promote more equitable pain assessment by supporting clinical decision-making when self-report is limited, inconsistent, or unavailable.
- Piq_β_ provides a consistent, standardized method for capturing physiological correlates of pain. It does not assume uniform pain experiences but ensures methodological consistency, enabling comparison while respecting their inherent variability.

**Abstract:**

(1) Background: Pain assessment still relies primarily on subjective self-report. To address these limitations, we developed Piq, an EEG-based index derived from beta-band brain activity (Piq_β_) aimed at providing objective pain identification and quantification. (2) Methods: The study combined cross-sectional and longitudinal designs. Resting-state brain activity was recorded for five minutes, and EEG signals were preprocessed using a dedicated algorithm. Piq_β_ performance was assessed by identifying an optimal cutoff to discriminate pain from no pain, evaluating its association with VNRS, and estimating agreement using a modified concordance criterion (exact match or ±1 category). A graded scale was also established to classify pain into distinct categories, according to intensity. (3) Results: An optimal cutoff of 10% for Piq_β_ yielded 97.8% sensitivity and 88.2% specificity. Piq_β_ correlated with self-reported scores (ρ = 0.60, *p* < 0.0001) with acceptable agreement (mean bias −1.02), accounting for clinically acceptable discrepancies. Five levels of pain were proposed, and Piq_β_ demonstrated the ability to track intra-individual fluctuations over time, accounting for clinically acceptable discrepancies. (4) Conclusions: These findings provide strong evidence to support the Piq_β_ index as a valuable complement to subjective pain ratings.

## 1. Introduction

Pain remains a major public health concern, posing persistent challenges in both its assessment and management. A robust and reliable evaluation is a critical prerequisite for any effective medical intervention. It is therefore not surprising that an estimated 20% to 30% of the global population experience some form of chronic pain [1], often leading to long-term consequences such as repeated navigation through fragmented care systems and increased risk of pharmacological dependence. Additionally, inadequate pain control is associated with a range of adverse outcomes, including increased morbidity and mortality, hospital readmissions, prolonged length of stay, and elevated healthcare system costs [2].

The definition of pain shows that it is both personal and complex, involving physical, emotional, and mental aspects [3,4]. Most healthcare professionals and researchers still use self-report tools—like the Visual Analog Scale (VAS) or Numeric Rating Scale (NRS)—to find out how much pain someone is feeling. These tools are useful, but they have clear limits: They rely on what the person says, which can be difficult for people who have trouble communicating, such as older adults or those with cognitive impairments [5,6]. The inability to measure pain objectively and within a reproducible framework limits both the accurate evaluation of pain and progress in translational research. Importantly, a reproducible model does not imply identical results across individuals, but it ensures consistent measurement conditions, which is essential for interpreting variability with confidence.

To promote a more consistent and objective assessment of pain, we developed the Piq (Pain Identification and Quantification) method, which incorporates the Piq_β_ index, a percentage-based metric quantifying the intensity of pain-related brain activity within the beta frequency band [7]. Piq’s procedures employ electroencephalography (EEG) to record ongoing cortical activity. EEG signals are then processed through an analytical pipeline that combines advanced methods and a custom algorithm based on the Hilbert transform to detect dynamic modulations in cortical activity associated with pain [8,9]. In the specific context of this study, cortical activity associated with pain refers to oscillatory changes arising from motor-related cortical regions, including the primary motor cortex (M1). Pain is known to modulate beta-band activity in these regions, reflecting motor inhibition and the integration of nociceptive information into motor output [10,11]. Because Piq captures the moment-to-moment variability of beta-band activity, it is theoretically well aligned with these pain-related modulations and provides a quantitative measure of their expression in cortical dynamics.

Unlike self-report tools, Piq provides an objective and consistent way to assess pain, independent of individual differences. It applies the same EEG parameters, uses a standardized five-minute window of EEG signals, and processes the data with the same algorithm to compute the Piq_β_ index, which reflects pain magnitude [7]. This ensures reliable, repeated measurements across both longitudinal and cross-sectional contexts, offering a standardized and non-invasive framework for quantifying pain intensity. Pain intensity results from the dynamic interplay of biological, psychological, and sociocultural factors. Elements such as genetic predisposition, emotional state, past experiences, attentional focus, cultural norms, and environmental context all modulate how pain is perceived and expressed [12,13]. Moreover, even with a standardized and reproducible assessment framework, results can fluctuate from one moment to the next within the same individual, further highlighting the inherent complexity of this condition. Due to the multifactorial nature of pain, individuals do not report it in the same way, even when clinical presentations appear similar. This is why each individual serves as their own reference point, thereby facilitating the interpretation of intra-individual variations over time.

Over the past two years, we have continuously tested the Piq procedure to assess its reproducibility across different contexts and user profiles. Importantly, this consistency refers to the validity and predictability of the relationship between the Piq index and the actual state of pain, not to the stability of pain itself, which is naturally dynamic [14]. As part of this effort, we have progressively improved the Piq algorithm and evaluated its performance across a range of experimental settings. In healthy volunteers exposed to thermal stimuli, the Piq_β_ index closely mirrored variations in self-reported pain intensity [7]. In collaboration with a veterinary team from the Université de Montréal, we applied Piq in a cross-sectional study to quantify pain in both awake and sedated companion animals. We observed a good performance of the Piq index, with strong correlations to a peripheral sensitization marker, which is considered a key indicator of nociceptive system responsiveness [15]. We also evaluated the ability of Piq to quantify pain in individuals living with pain conditions [7]. Across these varied contexts, the results have consistently been promising, demonstrating the robustness and adaptability of the Piq approach.

This article aims to present new key findings from both cross-sectional and longitudinal investigations conducted in individuals with and without pain in real-world settings outside the laboratory. The specific objectives of this study were two-fold: (1) To evaluate the performance of the Piq method in the identification and quantification of real-life pain, and (2) to assess its capacity to monitor intra-individual variability in pain over time. This study fits within the scope of the Special Issue ‘Trends in Chronic Pain Assessment and Treatment: Essential Aspects for Rehabilitation and Clinical Management’ by introducing an EEG-based brain index (Piq_β_) that complements traditional subjective measures. By offering a reproducible and objective assessment framework, our approach responds to the urgent need for innovative tools to support clinical decision-making and promote personalized management of chronic pain.

## 2. Materials and Methods

### 2.1. Study Design and Sample

This study combined two complementary methodological designs: An exploratory longitudinal design and a validating cross-sectional design. A total of 155 EEG recordings were collected from 48 individuals aged 23 to 76 years, including 40 participants with pain (23 females) and 8 without pain (6 males) (Table 1). Of these, 45 EEG recordings were obtained in a single session. A longitudinal design was applied to 3 participants. Two participants (1 female and 1 male) were observed twice daily—morning and afternoon, excluding weekends—over a period of 20 consecutive days. One participant (female) was monitored on the same schedule for 15 consecutive days. In total, the longitudinal study generated 110 EEG recordings. Participants were recruited through a combination of public announcements made during university research events, which were open to the community and allowed the recruitment of both individuals with chronic pain and healthy volunteers. Additional recruitment was conducted in collaboration with the regional pain association through their communication channels. Individuals who expressed interest contacted the research team and were screened according to the inclusion and exclusion criteria before being scheduled for an EEG recording session. The inclusion criteria for all participants were the ability to remain seated with eyes open for 10 consecutive minutes, and the capacity to provide a pain score every 30 s in response to an auditory beep over a 5 min period. Also, for ethical and clinical safety considerations, participants were not asked to discontinue their medications, including analgesics or neurological treatments. Participants with poorly controlled or uncontrolled epilepsy were excluded. In addition, participants wearing wigs were excluded because this interferes with proper electrode–scalp contact and compromises signal quality. The study received ethical approval from the institutional research ethics board (CER # 2024-1545 and CER # 2025-2029), and all participants provided written informed consent prior to any procedure. For pain identification, we aimed to determine the optimal cut-off value of the Piq_β_ index and to calculate its sensitivity and specificity as a binary detection tool. For pain quantification, we examined the association and agreement between Piq_β_ values and self-reported pain scores. Additionally, graded distribution of Piq_β_ values in correspondence to self-reported pain levels was proposed. The second specific objective focused on assessing the method’s ability to track dynamic changes in pain within individuals across repeated measures, thereby supporting its potential application in longitudinal pain monitoring.

### 2.2. Experimental Procedure

During data acquisition, participants remained seated at rest in a natural setting, with their eyes open and fixated on a black dot placed 1.5 m ahead at eye level. A wireless EMOTIV EEG headset was used to continuously record brain activity over a 5 min period (300 s). Pain intensity was recorded every 30 s (paced by a metronome beep) using a verbal numerical rating scale (VNRS) ranging from 0 (no pain) to 10 (worst imaginable pain) [16]. In the absence of within-session variability, repeated VNRS scores represent multiple measurements of the same underlying pain state. Averaging these ratings reduces random measurement error, increases reliability, and provides a more precise estimate of true pain intensity. This is the recommended psychometric approach when dealing with stable subjective constructs [17]. Accordingly, the mean VNRS value was computed to obtain a single representative pain score for the session, which was then correlated with the corresponding Piq value.

#### 2.2.1. EEG Signal Acquisition

**Figure 1:**
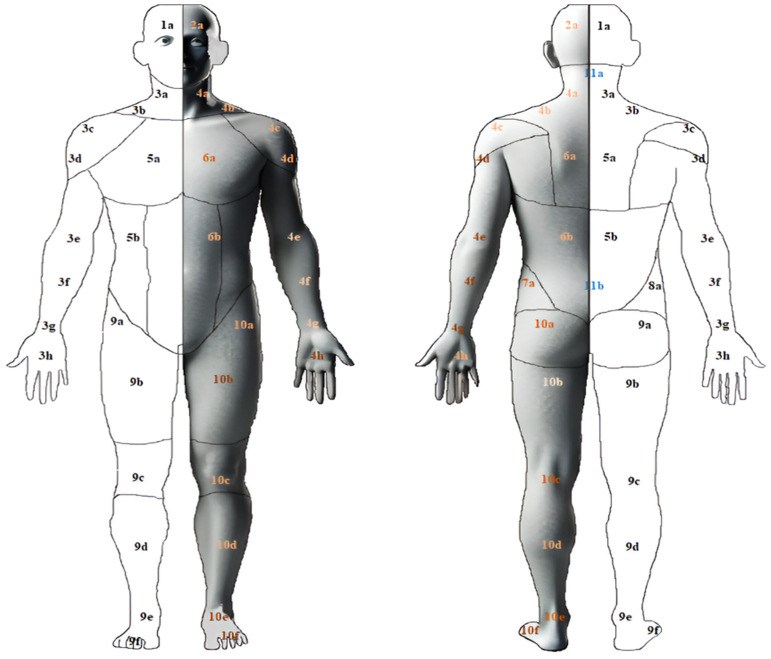
Body regions—electrode correspondence: Site 1—Right (zones: 9a, 9b, 9c, 9d, 9e, 9f) → Analyzed electrode: F3. Site 1—Left (zones: 10a, 10b, 10c, 10d, 10e, 10f) → Analyzed electrode: F4. Site 2—Right (zones: 1a, 3a, 3b) → Analyzed electrode: T7. Site 2—Left (zones: 2a, 11a, 4a, 4b) → Analyzed electrode: T8. Site 3—Right (zones: 3c, 3d, 3e, 3f, 3g, 3h, 5a, 5b, 11b, 8a) → Analyzed electrode: FC5. Site 3—Left (zones: 4c, 4d, 4e, 4f, 4g, 4h, 6a, 6b, 7a) → Analyzed electrode: FC6. The black color indicates the left hemibody, and the white color indicates the right hemibody. The blue color represents sites located at the mid-cervical and mid-lumbar spinal levels.

During the experimental protocol, EEG signal acquisition was performed using the Brainwear^®^ Emotiv EPOC X system (Emotiv Systems Inc., San Francisco, CA, USA), a wireless EEG headset equipped with 14 active electrodes and 2 reference electrodes, positioned according to the international 10–20 system. The recording channels are located at AF3, F7, F3, FC5, T7, P7, O1, O2, P8, T8, FC6, F4, F8, and AF4. Electrode contact quality was monitored in real time using a color-coded visual system: Each electrode had to appear green, indicating acceptable impedance levels (typically between 10 and 20 kΩ). This impedance was ensured by adequately moistening the electrode sponges with a saline solution and was continuously monitored throughout the EEG recording to ensure optimal signal quality. The headset uses a sequential sampling method with a single 16-bit analog-to-digital converter (ADC). EEG data were recorded at a sampling rate of 128 Hz. A built-in fifth-order Sinc digital filter was applied in real time, providing an effective bandwidth of 0.2–45 Hz, which covers the main EEG frequency bands (delta, theta, alpha, beta, and gamma). Additionally, integrated digital notch filters at 50 Hz (Europe) and 60 Hz (America) were applied to attenuate artifacts related to power line frequency interference. This real-time signal processing enhances EEG data quality. For each observation, one electrode from a subset of six electrodes of interest was selected for analysis: T7, F3, and FC5 on the left hemisphere, and T8, F4, and FC6 on the right hemisphere. Although these electrodes do not directly overlie canonical motor-homunculus sites, they represent the closest frontocentral positions available within the EMOTIV montage. These locations have been shown to capture distributed motor and nociception-related cortical activity, consistent with the involvement of frontocentral and sensorimotor regions in pain-related processing [18,19]. Wang et al. [20] also showed that EMOTIV’s configuration can reliably detect motor-related EEG patterns for both upper- and lower-limb movements, achieving 88.56% classification accuracy. This supports the ability of EMOTIV frontocentral electrodes to capture meaningful motor-related cortical signals despite the absence of canonical somatotopic coverage. For EEG analysis, electrodes were positioned according to the peripheral location of the pain to best reflect the corresponding cortical activity. When multiple pain sites were present, the most painful site was used as the reference for electrode placement. Electrode selection was guided by the participant’s reported pain location and its corresponding cortical representation. The electrode analyzed was contralateral to the side of pain: Right-sided pain was associated with electrodes over the left hemisphere (F3, T7, FC5), while left-sided pain corresponded to electrodes over the right hemisphere (F4, T8, FC6). Electrode positioning was based on the somatotopic correspondence between the body map (Figure 1) and the motor cortex. All healthy control participants were right-handed, so the same electrode location (FC5) was analyzed for all individuals. FC5 corresponds to the left frontal–central region, approximating the left primary motor cortex (M1), which includes the cortical representation of the right hand. This ensured that all control EEG recordings were processed using an identical cortical site and the same analytical pipeline as in the pain group.

#### 2.2.2. EEG Signal Preprocessing

After testing, EEG signal preprocessing was carried out following a rigorous five-step procedure, adapted from our previous work [7,21]. First, the direct current (DC) offset was removed to recenter the signal around its baseline. Second, artifacts related to eye blinks, movement, electromyographic (EMG) activity, or poor electrode–skin contact were identified and eliminated. The signal was then band-pass filtered to isolate the beta frequency band (13–30 Hz) for further analysis, as our previous studies have shown that beta activity is the most responsive and discriminative in relation to pain processing [7,21]. Next, a min–max normalization was applied to scale the filtered EEG signal to the [0,1] interval, facilitating comparison across conditions. Finally, a baseline normalization was performed by comparing the min–max normalized signal to a selected reference period. The full details of the preprocessing steps, including the methodological flowchart, have already been published in our previous article [7]. Following this preprocessing pipeline, the EEG signal was considered clean and standardized, and thus ready for subsequent analysis.

#### 2.2.3. Piq_β_ Index Computation

Following preprocessing, the free-artifact and normalized EEG signals were used to extract pain-related information encoded in brainwave activity. EEG signals reflect the combined electrical activity of multiple neuronal sources and naturally exhibit high complexity—often amplified in pain-related contexts [22,23]. To appropriately address this complexity, we employed a time–frequency analysis approach, focusing on variations in the EEG signal envelope. The envelope carries key information about signal energy, providing a simplified morphological representation of brain activity dynamics over time [24]. The Hilbert Transform (HT) is one of the most effective methods for extracting envelope-related features in EEG studies of pain [7,21,25]. EEG signals recorded from headsets are real-valued, meaning each point in time is represented by a single real number. By applying the HT, we obtain an analytic signal—a complex version of the original signal—that retains the same energy. This allows us to extract the signal’s instantaneous amplitude (its envelope), offering a clearer view of how brain activity changes over time in response to pain [24]. The Hilbert Transform approach is particularly effective in capturing the non-linear and non-stationary characteristics of brain activity [26], which are often overlooked by traditional techniques like the Fourier Transform that rely on assumptions of signal linearity and stationarity. Let $x_{\beta }\left(t\right)$ denote a real-valued EEG segment in the beta band (13–30 Hz). The first step involved extracting its imaginary component using the Hilbert Transform:

(1) $${\tilde{x}}_{\beta }\left(t\right)=HT\left\{x_{\beta }\left(t\right)\right\}=\frac{1}{\pi }p.v.\int_{-\infty }^{+\infty }\frac{x_{\beta }\left(\tau \right)}{\pi \left(t-\tau \right)}d\tau ,$$

where “p.v.“ refers to the Cauchy principal value, necessary to manage the singularity at t=τ. The analytic signal is then defined as:

(2) $$z_{\beta }\left(t\right)=x_{\beta }\left(t\right)+j.{\tilde{x}}_{\beta }\left(t\right)$$

The Hilbert Transform removes the redundancy of negative frequency components by preserving only the positive ones [27]. The signal envelope is computed as the magnitude of the analytic signal:

(3) $$E_{\beta }\left(t\right)=\sqrt{{x_{\beta }\left(t\right)}^{2}+{{\tilde{x}}_{\beta }\left(t\right)}^{2}}$$

The energy of this envelope is exactly twice that of the original signal, which ensures a complete energetic representation of brain activity [24]. In the fourth step, the dynamic variations in the envelope are analyzed using the coefficient of variation (CoV_β_), computed within sliding windows of 1 s, as follows:

(4) $${CoV}_{\beta }\left(t\right)=\frac{std\left(E_{\beta }\left(t\right)\right)}{Mean\left(E_{\beta }\left(t\right)\right)}\times 100,$$

where std and mean represent the standard deviation and the mean of the envelope values within the window, respectively. Finally, the average CoV_β_ over all windows is calculated, resulting in a global index of pain identification and quantification, denoted as Piq_β_ (%).

(5) $${Piq}_{\beta }\left(\%\right)=mean\left({CoV}_{\beta }\left(t\right)\right)$$

In our empirical observations, Piq_β_ (%) values below 10% typically reflect little or no pain, consistent with more synchronized and inhibited cortical activity. Conversely, values above 10% are generally associated with the presence of pain, reflecting cortical desynchronization and increased neuronal excitability [7,21]. The higher the Piq_β_ value (%), the greater the pain intensity.

### 2.3. Statistical Analysis

Given that the distributions of both variables of interest, VNRS pain scores and Piq_β_ (%), violated normality assumptions, a Shapiro–Wilk test performed on the dataset confirmed significant departures from normality (pain: W = 0.94, *p* = 3.8 × 10^−6^; Piq_β_: W = 0.95, *p* = 2.2 × 10^−5^). Moreover, the non-linear and non-stationary properties of EEG signals, combined with the multifactorial nature of self-reported pain intensity scores, further support the use of non-parametric statistical methods as the most appropriate analytical approach. The dataset comprises 155 observations, including data from the 3 participants involved in the longitudinal design. A summary of this data is presented in Table A1 (Appendix A). (1) To address specific objective 1—evaluating the performance of the Piq method in the identification and quantification of pain—the following analytical approach was undertaken:

#### 2.3.1. Pain Identification

(a) An empirical threshold of 10% for Piq_β_ had previously emerged from earlier datasets as a potentially meaningful boundary between pain and no-pain states. To formally validate this observation, the Youden index (J) was employed to identify the optimal cutoff value that maximizes the discriminative capacity of Piq_β_. This approach integrates both sensitivity and specificity, providing a robust criterion for selecting the threshold that best separates individuals experiencing pain from those who are not.
(b) The sensitivity and specificity of the Piq_β_ index were evaluated to determine its performance as a binary classifier for pain detection. Sensitivity was defined as the proportion of true pain cases correctly identified by Piq_β_ values above the threshold, while specificity referred to the proportion of pain-free cases correctly classified as such. These metrics were calculated based on the optimal cutoff identified via the Youden index.

#### 2.3.2. Pain Quantification

(a) Spearman’s rank correlation was employed to evaluate the association between self-reported pain intensity scores and the Piq_β_ index. This non-parametric method is well suited for capturing monotonic relationships in data that may be non-linear or influenced by individual variability. Its use supports the quantification of pain by establishing a statistically significant link between subjective pain reports and EEG-derived cortical signals.
(b) The Bland–Altman method was used to evaluate the agreement between two measures, including accuracy (bias), precision (SD), and limits of agreement (bias ± 1.96 × SD) [28]. The agreement performance of the Piq_β_ index was considered acceptable when both bias and precision remained below 2 points, as recommended in pain-related validation studies [29,30].
(c) A graded distribution of Piq_β_ values in relation to self-reported pain intensity scores was constructed by stratifying the 155 observations according to predefined VNRS categories. In line with multiple reports showing that optimal VNRS cut-off points for categorizing pain intensity may vary depending on patient characteristics [29,31,32,33], and supported by our own clinical experience, we adopted a stratification of self-reported pain scores into clinically meaningful categories (see Table 2: Pain score (0–10) categorization). The distribution of Piq_β_ values within each VNRS category was summarized using boxplots. Each boxplot displays the median and interquartile range (IQR), with whiskers extending to 1.5 times the IQR, allowing the identification of potential outliers in Piq_β_.

To address specific objective 2, which aimed to evaluate Piq_β_’s ability to reflect intra-individual fluctuations in pain across time, we combined visual trend analysis with complementary statistical methods to assess concordance between self-reported pain and EEG-based measures. Analyses were restricted to the first 15 days to ensure a consistent observation window across participants. First, to better account for individual variability in pain-related EEG activity, a subject-specific calibration of the Piq_β_ index was performed. For each participant, raw Piq_β_ values were rescaled to a 0–10 range based on their own minimum and maximum values. This normalization allowed for alignment between EEG responses and each participant’s subjective pain perception profile. Agreement between calibrated Piq_β_ values and self-reported scores was then assessed separately for AM and PM sessions. Following calibration, continuous error metrics were calculated using two standard indicators: Mean Absolute Error (MAE) and Root Mean Squared Error (RMSE), to evaluate the precision of the EEG-based index relative to self-reported pain. To further assess intra-individual agreement, we conducted Bland–Altman analyses using the calibrated ${Piq}_{\beta }$ values. For each participant and session (AM and PM), we examined whether the mean bias was minimal and whether all values remained within the limits of agreement, to determine the consistency between the EEG-based index and subjective pain reports. Agreement between VNRS and Piq was evaluated using a modified version of Cohen’s kappa statistic, in which concordance was defined as either exact agreement or ratings differing by no more than one category (within ±1). This approach accounts for clinically acceptable discrepancies between the two pain scales.

All signal processing and statistical analyses were performed using MATLAB (version 9.10.0.1602886, R2021a; MathWorks Inc., Natick, MA, USA), with the significance threshold set at *p* < 0.05.

## 3. Results

### 3.1. Patients’ Profile

**Longitudinal study participant profiles**

**Participant P1** was a former army veteran living with chronic pain for 204 months (17 years). All joints of the left lower limb were reported as painful, with the most intense and disabling pain located at the left ankle, according to the participant. Regular medications included Pregabalin (Lyrica^®^), an antiepileptic drug commonly used to treat neuropathic pain, and Oxycodone (OxyContin^®^), a strong opioid analgesic, was taken regularly or on demand. Morning medication was typically taken around 5:00 a.m., with EEG data collection occurring approximately four hours later, at 9:00 a.m. Afternoon EEG recordings were conducted around 3:00 p.m., approximately 10 h after the morning medication, with the next medication intake usually occurring afterward, around 5:30 p.m.

**Participant P2**, a veteran of the Canadian Air Force, who had served for 35 years. She had been retired for 8 years and had been experiencing pain for the past 120 months (10 years). The pain was primarily localized in the neck–shoulder region, head, and lower back. According to the participant, neck pain was the most persistent, intense, and disabling. On-demand analgesics consisted mainly of Tramadol, complemented by occasional use of supportive agents (e.g., anti-nausea and sleep/alternative therapies). Morning EEG data collection was performed around 10:00 a.m., typically 30 min before medication intake, which occurred around 10:30 a.m. Afternoon recordings were conducted at 2:00 p.m., approximately 3.5 h after the morning dose. Evening medications were generally taken at around 7:30 p.m.

**Participant P3** was a civilian and had been retired for 10 years. She had worked for 35 years as a workforce training director for multinational industries. She was diagnosed with stage 2 Parkinson’s disease in 2020 and had been experiencing chronic pain for the past 6 months. The pain was bilaterally localized in the hips, thighs, and lower back; however, according to the participant, the pain in both hips was significantly more intense and debilitating than the symptoms related to Parkinson’s disease. Her regular pain management included Gabapentin, taken three times daily (8:00 a.m., 4:30 p.m., and 10:00 p.m.), and topical Diclofenac (Voltaren^®^) cream used as needed. For Parkinson’s disease, she took an association of Levodopa + Benserazida (Prolopa^®^) at 12:00 p.m., 6:00 p.m., and 10:00 p.m. EEG data were collected at 10:00 a.m. and 2:00 p.m., corresponding to 2 h and 6 h, respectively, after her morning dose of Gabapentin.

### 3.2. Objective 1: Evaluating the Performance of ${Piq}_{\beta }$ for Pain Identification and Quantification

#### 3.2.1. Identification of Pain

##### The Optimal Cutoff Value of the Piq_β_ (%) Index

The optimal cutoff value of Piq_β_ (%) was determined to maximize discrimination between pain and no-pain conditions.

**Figure 2:**
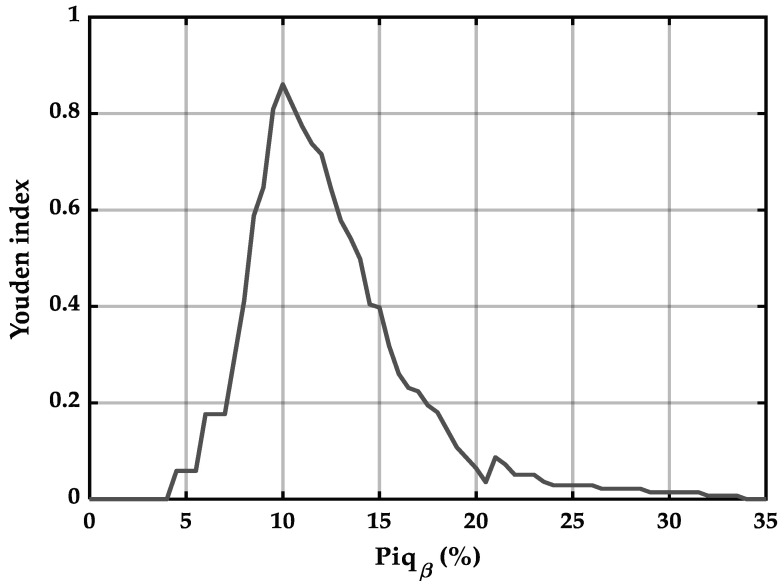
Optimal threshold for Piq_β_.

Figure 2 presents the Youden index (J) as a function of Piq_β_ index values. A Piq_β_ threshold of exactly 10% yielded the highest Youden index (0.86), confirming it as the optimal value for discriminating between the presence and absence of self-reported pain (score ≥ 1), with the best balance between sensitivity and specificity.

##### Sensitivity and Specificity of the Piq_β_ Index

Calculating sensitivity and specificity enables a comprehensive assessment of the index’s performance in the identification of pain; that is, its ability to correctly detect pain when present (sensitivity) and to correctly exclude it when absent (specificity). These metrics are thus used within a binary classification framework focused on pain identification. High values for both metrics, when combined with appropriate thresholds for the variables under study, strengthen the reliability of pain identification and support the validity of the classification model for pain and no-pain states. The Youden index (J) integrates sensitivity and specificity into a single metric of overall performance for binary classification. Table 3 presents the confusion matrix used to assess the discriminatory accuracy of the Piq_β_ index.

Sensitivity and specificity calculated:

- Sensitivity: 97.8%, Piq_β_ correctly detects pain in 97.8% of cases.
- Specificity: 88.2%, in 88% of cases, Piq_β_ does not signal pain when there is none.

#### 3.2.2. Quantification of Pain

##### Association Between Piq_β_ Index Values and Self-Reported Pain Scores

**Figure 3:**
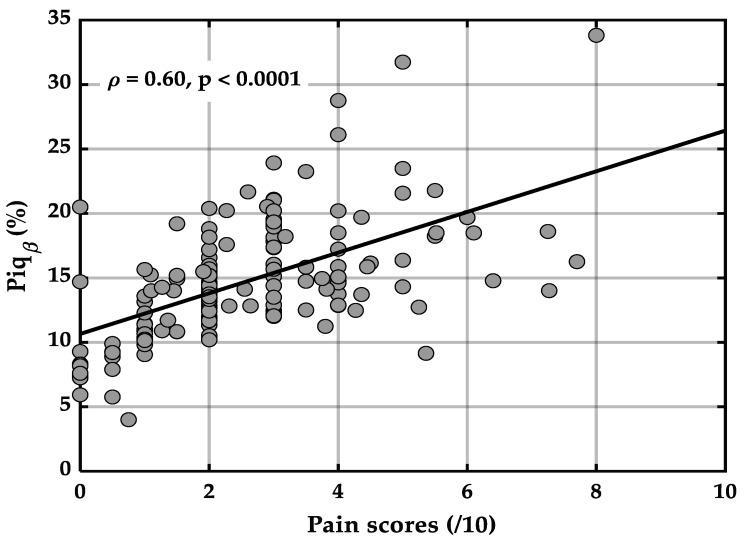
Association between self-reported pain and the Piq_β_ index.

The Spearman correlation coefficient revealed a moderate, positive, and statistically significant correlation between the Piq_β_ index and self-reported pain scores (Figure 3) (ρ = 0.60, *p* < 0.0001).

##### Agreement Between Piq_β_ Index and Self-Reported Pain Scores

**Figure 4:**
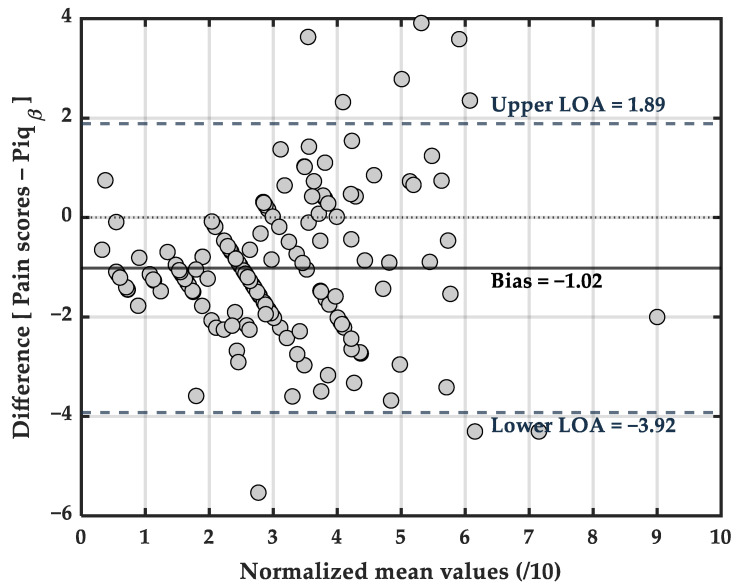
Agreement between Piq_β_ and self-reported pain scores.

This graph (Figure 4) displays a Bland–Altman agreement analysis, applied to data rescaled to a 0–10 range via Min–Max normalization, to assess the level of agreement between two pain assessment methods. The Limits of Agreement (LOAs) represent the interval within which 95% of the differences between the two measurement methods are expected to fall. They are calculated as: LOA = bias ± 1.96 × standard deviation of the differences. In this analysis, 95% of the differences between the two measures fell between –3.92 (Lower LOA) and +1.89 (Upper LOA) points. Piq_β_ index values were generally higher than self-reported pain scores. This accounts for the negative bias of −1.02, indicating that, on average, self-reported pain scores were approximately 1 point lower than those estimated by the Piq_β_ index. The standard deviation (SD) of the differences was 1.48, reflecting acceptable precision between the two methods.

##### Proposed Graded Scale for Piq_β_

**Figure 5:**
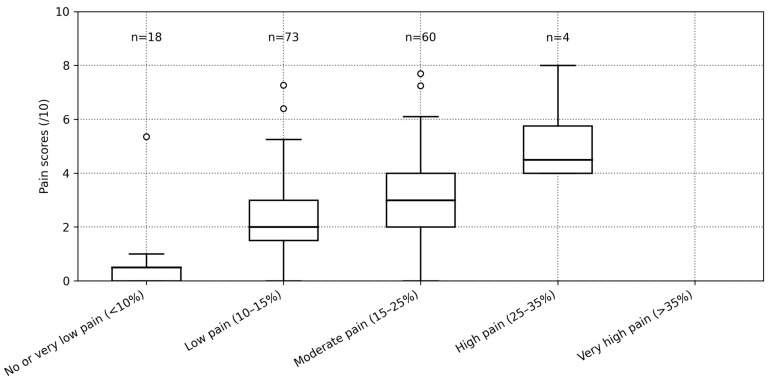
Proposed graded distribution of Piq_β_ values in correspondence to self-reported pain levels (n = 155): Boxes represent medians and interquartile ranges; whiskers extend to 1.5× the interquartile range (IQR). Values beyond this range are plotted as individual points and considered statistical outliers.

As a reminder, pain intensities were stratified according to thresholds supported by previous literature and further refined based on our clinical experience, ensuring that the proposed categories reflect both empirical evidence and practical relevance. Piq_β_ values were then mapped onto these pain categories (Figure 5).

Categories were defined as follows: No or very low pain (<10%), Low pain (10–15%), Moderate pain (15–25%), High pain (25–35%), and very high pain (>35%). Sample sizes (n) are displayed above each boxplot. Horizontal dashed lines in the boxes indicate Piq_β_ thresholds (10%, 15%, 20%, 25%, 30%, and 35%). Outliers were identified using the 1.5 × IQR rule applied to pain scores within each Piq_β_ category. This procedure revealed one outlier in the No or very low category, two in the Low category, and two in the Moderate category, with pain scores ranging from 5.36 to 7.70. The distributions showed consistent internal progression. Interquartile ranges were relatively narrow, indicating good internal consistency of Piq_β_ values within each category. Table 2 summarizes the distribution of Piq_β_ values and corresponding self-reported pain scores across the five proposed pain categories.

**Figure 6:**
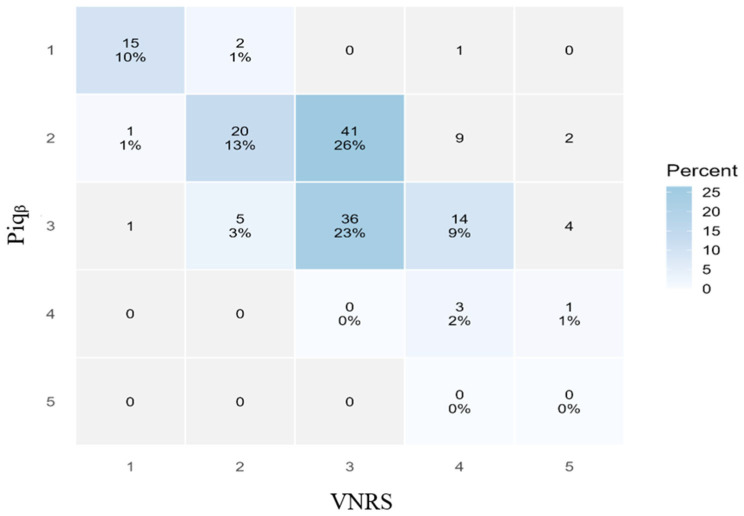
Heat map of agreement between VNRS and Piq_β_ categories. Each cell represents the number and percentage of paired observations. Darker blue shades indicate higher frequencies. Exact concordance is shown on the diagonal, while adjacent cells (±1 category) are considered clinically acceptable agreement.

Most observations fell within the low (n = 73) and moderate (n = 60) categories, while no cases were classified in the very high pain category (Piq_β_ > 35%). When considering concordance within ±1 category, the agreement was moderate (κ = 0.52), supporting that small discrepancies between VNRS and Piq are clinically acceptable, according to Landis and Koch (1977) [34]. To visualize the pattern of agreement between VNRS and Piqᵦ, we constructed a heat map of the 5 × 5 contingency table. The intensity of the color scale reflects the number of paired observations, allowing an immediate assessment of both exact agreement (diagonal) and the distribution of disagreements across categories (Figure 6).

Table 4 summarizes the main results related to the performance of the Piq_β_ index. 

#### 3.2.3. Use of the Piq_β_ Index in a Longitudinal Design

While rescaling Piq_β_ to a 0–10 scale was useful to illustrate its correspondence with self-reported pain scores during analysis, it is important to emphasize that Piq_β_ is fundamentally designed to be expressed as a percentage. This format reflects the relative proportion of cortical activity associated with pain, making it scalable for intra-individual follow-up, inter-session comparisons, and practical clinical use. Keeping Piq_β_ in its original percentage form also ensures ease of interpretation and consistency with its intended role as a physiological marker. Therefore, although normalization may support validation efforts during development, the percentage format should be preserved in practical applications.

- Results for Participant 1

**Figure 7:**
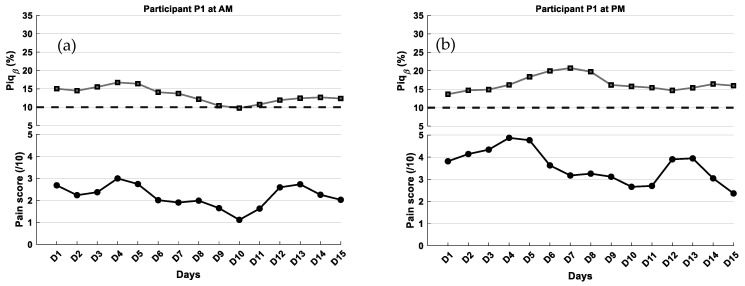
Self-reported pain scores and Piq_β_ index within the same individual (P1) over a 15-day period: (**a**) Morning (AM) and (**b**) afternoon (PM) sessions. The curves were smoothed using a fourth-order Savitzky–Golay filter. The black dashed line represents the 10% Piq_β_ threshold indicating the presence of pain.

For participant P1, morning sessions were associated with low self-reported pain scores (mean ≈ 2) and Piq_β_ values predominantly below 13% (Figure 7a), both suggesting low pain intensity according to our proposed Piq_β_ grading scale. In the afternoon sessions, pain intensity increased to a moderate level, with a mean self-reported score of approximately 3.5 and Piq_β_ values around 15% (Figure 7b), aligning with the moderate pain category on the same scale. The subject-specific calibration of the Piq_β_ index yielded moderate agreement with self-reported pain scores over the first 15 days. The mean absolute error (MAE) was 2.14 in the AM and 2.44 in the PM, while the root mean squared error (RMSE) was 3.03 and 2.96, respectively.

A Bland–Altman analysis was also performed using the calibrated Piq_β_ values. In the AM session, the mean bias was −2.01, and one value falling outside these limits. In the PM session, the mean bias was −0.33, and one outlier was observed. These findings suggest a more stable agreement between subjective pain scores and the EEG-based index in the afternoon. As a reminder, EEG data were collected approximately 4 h after the morning medication for the AM session, and about 10 h later for the PM session. The participant’s regular medications included Pregabalin, an antiepileptic agent used to treat neuropathic pain, and OxyContin, a strong opioid analgesic.

- Results for participant 2

**Figure 8:**
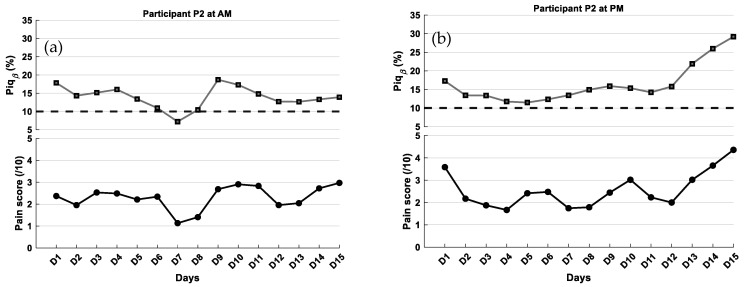
Self-reported pain scores and Piq_β_ index within the same individual (P2) over a 15-day period: (**a**) Morning (AM) and (**b**) afternoon (PM) sessions. The curves were smoothed using a fourth-order Savitzky–Golay filter. The black dashed line represents the 10% Piq_β_ threshold indicating the presence of pain.

For participant P2, morning sessions were characterized by low pain intensity, with self-reported scores averaging 2 and mean Piq_β_ values close to 14% (Figure 8a), both corresponding to the Low pain category on the proposed Piq_β_ scale. In the afternoon, a slight increase in pain was observed, with average self-reported scores nearing 3 and Piq_β_ values around 16% (Figure 8b), consistent with Moderate pain. A strong agreement between the calibrated Piq_β_ index and self-reported pain scores was observed for Participant 2. The mean absolute error (MAE) was 1.87 in the AM and 1.60 in the PM, while the root mean squared error (RMSE) was 2.34 and 2.10, respectively. To further assess intra-individual agreement, a Bland–Altman analysis was performed using the calibrated ${Piq}_{\beta }$ values. In both AM and PM sessions, the mean bias was small (approximately −0.25), and all values fell within the limits of agreement. As a reminder, morning EEG data collection was typically performed about 30 min before medication intake, while afternoon recordings took place approximately 3.5 h after the morning dose. Her regular pharmacological treatment included an antihypertensive (Riva-Perindopril), a thyroid hormone replacement (Synthroid), and anti-inflammatory and analgesic agents (Celecoxib and acetaminophen).

- Results for participant 3

**Figure 9:**
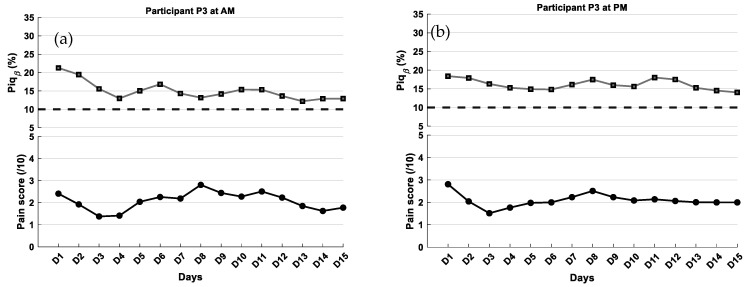
Self-reported pain scores and Piq_β_ index within the same individual (P3) over a 15-day period: (**a**) Morning (AM) and (**b**) afternoon (PM) sessions. The curves were smoothed using a fourth-order Savitzky–Golay filter. The black dashed line represents the 10% Piq_β_ threshold indicating the presence of pain.

For participant 3, in both morning and afternoon sessions, the graphs show that average self-reported pain scores were approximately 2, indicating moderate perceived pain. Mean Piq_β_ values were 15% (Figure 9a) and 16% (Figure 9b), respectively, suggesting a moderate level of pain intensity according to the proposed Piq_β_ scale. For Participant 3, the subject-specific calibration showed less precise alignment between Piq_β_ and self-reported pain scores. The MAE was 2.68 in the AM and 2.70 in the PM, with RMSE values of 3.45 and 3.53, respectively. Bland–Altman analysis indicated a consistent negative bias in both sessions (AM: −2.10; PM: −1.97), with wide limits of agreement (AM: −7.65 to +3.46; PM: −7.92 to +3.99) and no values falling outside. As a reminder, EEG data were collected approximately 2 h (AM) and 6 h (PM) after her Gabapentin dose.

## 4. Discussion

Performance of the Piq_β_ index for pain identification and quantification: This study provides strong empirical support for the use of the Piq_β_ index as a valid, objective measure of pain intensity based on EEG signal dynamics. The core contribution of this work lies in demonstrating that the Piq_β_ index can accurately identify the presence of pain, quantify different levels of pain intensity, and respond to fluctuations in pain within the same individual over time.

Determination of the optimal Piq_β_ cutoff value and associated sensitivity and specificity for the identification of pain: The first piece of evidence supporting the empirical validity of the 10% threshold for pain detection using Piq_β_ lies in the identification of an optimal cut-off indeed located at 10%, with a maximal Youden index of 0.86. This cut-off, corresponding to self-reported pain scores equal to or greater than 1 on a 0 to 10 scale, yielded a sensitivity of 97.8% and a specificity of 88.2%, demonstrating that Piq_β_ performs remarkably well in detecting true pain states while minimizing false positives. These results on the sensitivity and specificity of Piq_β_ at the identified thresholds (self-reported pain ≥ 1; Piq_β_ ≥ 10%) are in line with a recent EEG-based pain detection study, in which a system using convolutional and recurrent neural networks achieved accuracies of ~91.8% for pain detection and 87.9% for severity classification [22]. Although that study did not report error metrics such as MAE or RMSE, its high classification accuracy suggests strong model performance, comparable to the detection capabilities observed with the Piq_β_ index in this study. Together, these findings suggest that the Piq_β_ index holds discriminative potential for identifying the presence of pain, combining high sensitivity with acceptable specificity. The convergence between empirical trends, optimal threshold determination, and external benchmarks reinforces its promise as a neurophysiologically informed indicator for pain detection.

Quantification of pain using the Piq_β_ index: A central aim of this study was to evaluate the performance of the Piq_β_ index to quantify pain intensity in a physiologically grounded and methodologically reproducible manner. This objective was addressed through a two-pronged approach: Assessing the association between cortical signals and self-reported pain scores and quantifying the level of agreement between both methods across repeated measures in a longitudinal design. A moderate and statistically significant positive association was observed between Piq_β_ values and self-reported pain intensity scores, reinforcing the index’s potential for pain quantification (Figure 3). Although the two measures are not perfectly aligned—a discrepancy expected due to the inherently subjective nature of pain and the influence of biopsychosocial and contextual factors on self-reports—the observed correlation coefficient (ρ = 0.60) supports the capacity of the Piq_β_ index to reflect fluctuations in pain intensity through cortical activity. This level of association supports the idea that the index encodes meaningful cortical dynamics underlying pain perception.

To further evaluate the accuracy of pain quantification by the Piq_β_ index, a Bland–Altman analysis was conducted using min–max normalized values on a 0–10 scale as part of a calibration procedure to align Piq_β_ values with self-reported pain ratings (Figure 4). The result showed a mean bias of −1.02 on a 0–10 scale, with 95% limits of agreement ranging from −3.92 to +1.89 and the standard deviation of the difference (SD = 1.48). For instance, in blood pressure monitoring, a mean bias of ±4–5 mm Hg with a standard deviation ≤ 8 mm Hg is generally accepted between manual and automated devices, summarized in terms as ≥85% of paired readings within ±10 mmHg and ≥95% within ±15 mmHg between manual and automated devices [35]. In glucose monitoring, a bias of ±15% is often tolerated between capillary and venous glucose readings, with acceptable SD ranging from 5 to 10% depending on the context [36,37]. For body temperature, differences of up to ±0.5 °C are commonly accepted between contact digital thermometers and infrared forehead thermometers, corresponding to a mean bias of around ±2–3% and SD typically under 0.3 °C [38]. These analogies underscore the robustness of the ${Piq}_{\beta }$ index for pain quantification, considering its observed bias, limits of agreement, and standard deviation, all of which fall within the acceptable ranges for physiological measurement tools.

A graded interpretation scale based on the Piq_β_ index: Moreover, a five-tiered graded interpretation scale based on ${Piq}_{\beta }$ values was derived from the empirical distribution of 155 observations and their corresponding self-reported pain scores. This data-driven categorisation aims to enhance internal coherence between physiological signals and subjective reports. The resulting scale—“no or very low pain” (<10%), “low pain” (10–15%), “moderate pain” (15–25%), “high pain” (25–35%), and “very high pain” (>35%)—offers a practical framework for monitoring pain dynamics over time. This structured categorization not only improves interpretability but also provides a standardized language for Piq-based pain quantification. These categorizations were supported by narrow interquartile ranges (IQRs) and few statistical outliers, reinforcing the internal consistency of the Piq_β_ graded scale [39]. In our dataset of 155 EEG observations from individuals living with pain, most values fell within the “low” (41.3%) and “moderate” (33.5%) categories. This distribution aligns with previous findings on pain fluctuations in individuals with chronic pain [11,40]. The IQRs were relatively narrow: 2.5% (11.3–13.8%) for the “low pain” category and 6.9% (16.0–22.9%) for the “moderate pain” category. While there is no fixed rule for IQR thresholds, such low dispersion (IQR < 30%) is generally seen as desirable [41]. In human biomarker research, the coefficient of variation is commonly used, and values below 10% are generally accepted as indicators of acceptable intra-subject variability [42,43]. This contextual benchmark reinforces the interpretation of our narrow IQRs as indicators of consistency and stability in the stratified output of the Piq_β_ scale.

Responsiveness of Piq_β_ index to pain fluctuations in longitudinal monitoring: One of the most innovative aspects of this study lies in the application of the Piq_β_ index within a longitudinal design. Beyond cross-sectional measurement, the objective here was to evaluate whether Piq_β_ could reliably track intra-individual fluctuations in pain intensity over time. The findings are promising across the three participants assessed over two sessions (AM and PM), as the temporal dynamics of Piq_β_ values largely mirrored self-reported pain ratings. The longitudinal analysis revealed distinct participant profiles (Table 5). For Participant 1, Piq_β_ values rose from morning to afternoon in parallel with self-reported pain scores, reflecting a shift from the “low pain” to the “moderate pain” category on the proposed graded scale; agreement between measures was also stronger in the afternoon, with reduced bias and narrower error margins. Participant 2 showed the most consistent pattern, with Piq_β_ closely matching pain ratings across both sessions, showing minimal mean errors, and all observations falling within the Bland–Altman limits of agreement. By contrast, Participant 3 displayed the weakest alignment, as Piq_β_ diverged from reported pain scores, leading to the largest mean errors and the broadest limits of agreement. Specifically, the results show that participants P1 and P2 exhibited relatively low error values (MAE: 1.60–1.87; RMSE: 2.08–2.34), while participant P3 showed the highest errors in both sessions (MAE ≈ 2.70; RMSE > 3.45). These findings are consistent with previous studies reporting similar levels of performance for EEG-based pain assessment tools [44]. For instance, facial-expression-based approaches for automatic pain estimation under real-world conditions have shown MAEs up 2.0 [45], while EEG-based deep learning models have reported MAEs of 2.0–2.5 and RMSEs up to 4.0, depending on context and population [46]. The relatively low errors observed in participants P1 and P2 suggest a coherence that may stem from the absence of neurological conditions directly affecting cortical pain processing. In contrast, participant 3, who presented with Parkinson’s disease and a complex pharmacological regimen (including Gabapentin and dopaminergic therapy), exhibited the highest MAE and RMSE values. Despite stable self-reports pain scores, the Piq_β_ index showed reduced precision in estimating pain, possibly due to altered cortical oscillatory patterns and drug-induced modulation of pain circuits. These findings highlight the importance of individualized interpretation, whereby each subject serves as their own reference for monitoring pain dynamics [47]. Such a personalized approach accounts for neurophysiological heterogeneity across individuals and reinforces the practical value of using Piq_β_ as a longitudinal tracking tool, rather than relying solely on uniform thresholds applied to the entire population.

Another factor influencing Piq_β_ index performance was the timing of EEG acquisition relative to medication intake. In the longitudinal design, sessions were scheduled before or after analgesic administration, revealing that the most consistent agreement between Piq_β_ values and pain scores occurred when medication effects were present but not at their peak, reducing pharmacodynamic interference and yielding more stable cortical signals. For Participant 3, who showed the weakest agreement, this may be explained by overlapping pharmacokinetics and the known impact of Parkinson’s disease on cortical excitability. Interestingly, for P1, better agreement was found in the morning session, despite earlier medication intake, suggesting a potentially delayed or attenuated drug effect. These findings emphasize the need to optimize EEG acquisition timing to best capture cortical activity associated with pain, especially in the context of complex pharmacological regimens. Naturally, although our intention was to create three clinically homogeneous entities through the study design, the challenge of recruiting neurologically comparable participants remains inherent. These conclusions must therefore be interpreted with caution, given the small sample size.

Strengths and limitations: A key strength of the Piq_β_ index lies in the robust number of observations collected in the cross-sectional component, which included a diversity of pain types. This dataset enabled the identification of an optimal detection threshold and the quantification of pain with acceptable accuracy. For the Bland–Altman analysis, a priori power calculation indicated that at least 117 paired observations were necessary to achieve 95% statistical power to detect agreement within clinically relevant limits [48]. Moreover, the Piq method is firmly grounded in established neurophysiological theory, enhancing its interpretability and scientific validity. Specifically, Piq_β_ leverages the dynamic modulation of beta-band activity, a frequency range consistently implicated in sensorimotor integration and pain processing [49,50]. In addition, the use of the Hilbert transforms in the algorithm’s formulation, particularly its ability to extract EEG signal envelopes, enhances the Piq_β_ index’s sensitivity to fluctuations in cortical activity, while preserving signal integrity and ensuring interpretability [7]. In chronic pain conditions, a well-established shift is observed in the balance of inter-neuronal activity: Increased cortical excitability, driven by heightened facilitation, is frequently accompanied by diminished inhibitory tone [51]. This imbalance reflects maladaptive plasticity within the neural circuits involved in pain processing. Expressed as a percentage, it offers a quantifiable measure of the relative contributions of facilitation and inhibition to overall cortical excitability, this neurophysiological grounding makes Piq_β_ particularly valuable for monitoring the dynamic brain states associated with persistent pain. However, this study also has limitations. Mainly, the longitudinal component included only three individuals, yielding a limited number of observations. This small sample fell short of the statistical power required for robust agreement analysis using the Bland–Altman method, which limits the generalizability of the findings. To strengthen the longitudinal evidence, future studies should replicate these results in larger cohorts, with each participant representing a clinically homogeneous pain profile, whether experiencing chronic pain or not. Moreover, the data distribution was unbalanced, with a larger proportion of observations associated with the presence of pain (Table 3), which could influence certain global indicators such as overall accuracy. The relatively small number of pain-free cases may also impact and contribute to the reduced precision of specificity and negative predictive value estimates. To address this, increasing the number of pain-free cases would be a necessary potential solution. Because participants continued their usual medications, including analgesic and neurological treatments, potential drug effects on cortical oscillations cannot be fully excluded. However, our previous work indicates that analgesic medications tend to reduce Piq values rather than increase them, suggesting that medication use would bias the results toward underestimating, rather than exaggerating, pain-related cortical variability [7].

Finally, outliers in Piq_β_ were defined relative to VNRS categories, providing a preliminary framework to construct a graded pain scale. While this stratification offers a pragmatic reference attached in self-reported experience, it is important to note that Piq_β_ does not capture the same components as pain scores. Piq_β_ reflects functional neurobiological processes within the central nervous system, whereas VNRS scores encompass the broader and multidimensional experience of pain. As its use continues, this initial categorization of graded Piq_β_ scales may be further refined.

## 5. Conclusions

This study provides converging evidence supporting the performance of the Piq_β_ index as a valuable complement to subjective pain ratings. It demonstrated high sensitivity and specificity, low error margins, and reliable quantification of pain intensity. Beyond cross-sectional detection, the index showed promising responsiveness to intra-individual fluctuations in pain over time, reinforcing its applicability for longitudinal monitoring. Importantly, Piq_β_ retains interpretability through its grounding in cortical beta-band dynamics, offering a transparent and physiologically meaningful alternative to black-box approaches. Taken together, these findings position Piq_β_ as a promising tool that bridges objective neurophysiological data with the lived experience of pain. It contributes to the emergence of a shared interpretive framework, offering a common language for all stakeholders involved in the assessment, research, and management of pain. Moreover, this innovative approach could be particularly useful in situations where patients cannot reliably self-report their pain, such as in intensive care units, in individuals with cognitive impairment, or in those with speech difficulties following a stroke or other conditions. This represents a substantial contribution, as no objective tool currently exists to adequately address pain assessment in these challenging contexts. The Piq_β_ index offers clinicians and rehabilitation professionals an additional objective tool to enhance clinical management, particularly in contexts where self-report is limited or unreliable.

## 6. Patents

Ngomo, S. & Segning, C. Method and System for Pain Identification and Quantification Using Electroencephalogram Signals. Patent application pending, PCT/CA2024/050268–WO/2024/182890, 2024.

## Figures and Tables

**Table 1:** Participants’ characteristics.

	Longitudinal (n = 3)		Cross-Sectional—Chronic and Acute Pain (n = 37)			Cross-Sectional—Healthy Controls (n = 8)	
			Chronic Pain (n = 36)		Acute Pain (n = 1)		
Sex	Women (n = 2)	Men (n = 1)	Women (n = 21)	Men (n = 15)	Men (n = 1)	Women (n = 2)	Men (n = 6)
Age (years)	67.5 ± 7.7	45.0 ± 0.0	55.1 ± 14.5	54.9 ± 18.1	39.0 ± 0.0	36.5 ± 9.2	36.0 ± 19.3
Weight (Kg)	78.1 ± 4.38	108.0 ± 0.0	82.2 ± 20.7	89.3 ± 22.5	110.0 ± 0.0	79.5 ± 26.1	79.0 ± 13.2
Height (m)	1.57 ± 0.01	1.70 ± 0.0	1.64 ± 0.08	1.77 ± 0.07	1.70 ± 0.0	1.57 ± 0.02	1.73 ± 0.09
Body Mass Index (Kg/m^2^)	31.62 ± 2.23	37.37 ± 0.0	30.49 ± 8.02	28.34 ± 6.34	30.79 ± 0.0	31.91 ± 9.68	26.24 ± 3.21
Pain duration (months)	63.0 ± 80.6	204.0 ± 0.0	140.3 ± 119.9	242.3 ± 19.2.9	2.0 ± 0.0	0.0 ± 0.0	0.0 ± 0.0

Note. Data are presented as mean ± standard deviation (SD).

**Table 2:** Pain grading proposal based on Piq_β_.

Pain Classes Based on Piq_β_ Values	n (155)	Pain Score (0–10) Categorization	Proposed Piq_β_ (%) Thresholds	Number of Outliers Pain Score Values (n)	Outliers Pain Score Values
No or very low pain	18	<1	< 10	1	5.36
Low pain	73	1 ≤ score < 2	10–15	2	7.27 6.40
Moderate pain	60	2 ≤ score < 4	15–25	2	7.25 7.70
High pain	4	4 ≤ score < 6	25–35	0	
Very high pain	0	≥6	>35	0	

**Table 3:** Performance of Piq (Thresholds: Piq_β_ ≥ 10% and self-reported pain ≥ 1).

	Presence of Pain (≥1)	Absence of Pain (<1)
Piq_β_ detects pain (≥10%)	135 (TP) True positives	2 (FP) False positives
Piq_β_ does not detect (<10%)	3 (FN) False negatives	15 (TN) True negatives

**Table 4:** Overview of the main results related to the performance of the Piq_β_ index.

Performance for	Test	Result	Significance
Cut-off value for Piq_β_ (%): Pain identification	Youden index (J)	Youden index reaches maximum (0.86) at Piq_β_ = 10% and self-reported pain score ≥ 1	A 10% Piq_β_ threshold was determined to be the optimal cut-off for differentiating pain from no or low pain, offering the best trade-off between sensitivity and specificity for accurate pain detection.
Piq_β_ Sensitivity: Pain identification	J = Sensibility + specificity − 1	Sensitivity = 98.4%	Piq_β_ index correctly identified pain in 98.4% of individuals who reported a pain score ≥ 1.
Piq_β_ Specificity: Pain identification		Specificity = 88.2%	Piq_β_ correctly identifies the absence of pain in 88% of cases.
Pain quantification	Spearman correlation	ρ = 0.60 (*p* < 0.0001).	
	Bland–Altman (Piq_β_ normalized on a 10-point scale)	In 95% of cases, the difference between the self-reported pain score and Piq_β_ ranges from −3.92 to +1.89 points. Bias = −1.02: On average, participants report pain scores about 1 point lower than those estimated by Piq_β_.	
Pain quantification: Graded scale for Piq_β_ intensity	Categories were defined based on Piq_β_ values and validated using observed pain score distributions and outlier analysis (1.5 × IQR rule)	Proposed graded scale for Piq_β_ intensity ▪ None or very low pain if Piq_β_ < 10% ▪ Low pain if 10% ≤ Piq_β_ < 15% ▪ Moderate pain if 15% ≤ Piq_β_ < 25% ▪ High pain if 25% ≤ Piq_β_ < 35% ▪ Very high pain if Piq_β_ ≥ 35%.	

**Table 5:** Mean Prediction Errors (MAE and RMSE) across 15-day AM and PM sessions for each participant.

Participant	MAE		RMSE	
	AM	PM	AM	PM
P1	1.87	1.60	2.34	2.10
P2	1.63	1.60	2.11	2.08
P3	2.68	2.70	3.45	3.53

## Data Availability

The results derived from the EEG signals, including summary tables and statistical analyses, are fully reported in the manuscript. The raw data (EEG SIGNALS) are not publicly available due to confidentiality constraints and their inclusion in an active patent application. Public sharing would compromise intellectual property protection.

## Appendix A

**Table A1:** Descriptive statistics. Notes: T7/T8 = left/right temporal; FC5/FC6 = left/right frontocentral; F3/F4 = left/right frontal. Electrodes of interest were positioned over these areas, based on the peripheral location of the reported pain (most painful site).

Observations (ID)	Pain Score (/10)	${Piq}_{\beta }$	Sex: 1 = Female 2 = Male	Age (Years)	Pain Duration (Months)	Most Painful Site	Electrode of Interest	Pain Medication
1	3.00	16.06	2	44	204	Left ankle	F4	Yes
2	2.00	12.84						
3	2.00	16.57						
4	3.50	15.83						
5	3.00	19.22						
6	1.50	10.82						
7	2.00	15.89						
8	2.00	11.35						
9	2.00	10.44						
10	0.50	8.89						
11	1.50	10.83						
12	3.00	12.46						
13	3.00	12.15						
14	2.00	13.22						
15	2.00	12.00						
16	1.00	9.05						
17	0.50	5.76						
18	1.50	14.94						
19	1.50	19.20						
20	1.00	10.41						
21	3.50	12.51						
22	4.50	16.15						
23	4.00	13.75						
24	5.00	16.37						
25	5.50	18.23						
26	3.00	21.10						
27	3.00	19.54						
28	3.50	23.25						
29	3.00	12.21						
30	3.00	17.34						
31	1.50	15.20						
32	5.00	14.31						
33	4.00	14.77						
34	3.00	17.83						
35	2.00	15.16						
36	3.00	18.16						
37	0.50	9.27						
38	2.00	11.85						
39	1.00	9.82						
40	1.00	13.15						
41	3.00	21.03	1	62	120	Neck–shoulder	FC5/FC6	Yes
42	1.00	10.88						
43	3.00	15.12						
44	3.00	18.95						
45	1.00	11.03						
46	4.00	12.86						
47	0.00	5.93						
48	0.75	4.00						
49	4.00	28.76						
50	2.00	12.68						
51	4.00	17.23						
52	1.00	10.32						
53	2.00	13.44						
54	3.00	12.89						
55	3.00	14.40						
56	0.50	9.91						
57	2.00	14.65						
58	2.00	12.41						
59	1.00	11.44						
60	3.00	13.50						
61	5.00	21.57						
62	0.50	7.90						
63	3.00	17.39						
64	0.50	9.21						
65	3.00	12.01						
66	3.00	12.06						
67	1.00	13.59						
68	2.00	14.63						
69	2.00	17.19						
70	4.00	14.61						
71	2.00	15.23						
72	1.00	11.38						
73	4.00	26.11						
74	3.00	23.92						
75	5.00	31.74						
76	1.00	10.67						
77	4.00	15.88						
78	4.00	20.20						
79	1.00	10.25						
80	2.00	11.68						
81	2.60	21.67	1	73	6	Hips (bilateral)	F3/F4	Yes
82	2.00	20.39						
83	1.09	15.24						
84	1.27	10.90						
85	2.00	14.58						
86	3.00	20.21						
87	1.00	12.28						
88	4.00	12.89						
89	2.00	14.02						
90	2.00	15.98						
91	3.00	15.67						
92	2.00	14.19						
93	2.00	10.20						
94	1.27	14.27						
95	2.00	12.43						
96	3.18	18.21						
97	2.00	18.82						
98	1.00	15.65						
99	2.00	15.11						
100	2.00	15.04						
101	2.00	14.42						
102	2.00	15.48						
103	3.00	19.34						
104	2.00	15.70						
105	2.00	13.33						
106	2.27	20.22						
107	2.00	18.16						
108	2.00	13.76						
109	2.00	15.19						
110	2.00	13.54						
111	0.00	9.29	2	27	0	–	FC5	No
112	0.00	20.50	2	42	0	–	FC5	No
113	0.00	14.70	2	26	0	–	FC5	No
114	0.00	8.32	2	24	0	–	FC5	No
115	0.00	7.41	2	24	0	–	FC5	No
116	2.90	20.54	2	33	54	Right leg	F3	
117	3.50	14.74	1	35	60	Neck–shoulder	FC5/FC6	-
118	2.00	14.11	1	47	120	Lower back	F4	-
119	0.00	8.30	1	29	0	–	FC5	No
120	2.00	14.10	2	39	2	Lower back	F4	-
121	0.00	7.25	1	42	0	–	FC5	No
122	1.45	14.00	1	61	60	left shoulder	FC6	-
123	5.52	18.50	2	57	120	Lower back	F4	-
124	2.55	14.12	1	46	120	Lower back	F4	-
125	1.00	10.14	2	25	6	Knee (bilateral)	F3/F4	-
126	6.10	18.49	1	49	12	left shoulder	FC6	-
127	2.00	10.52	1	62	120	Left leg	FC6	-
128	3.82	14.12	2	23	120	Lower back	F4	-
129	7.27	14.01	2	29	7	Lower back	F4	-
130	4.00	18.50	1	46	48	Neck–shoulder	FC6	-
131	1.10	14.00	1	38	24	Left shoulder	FC6	-
132	2.00	13.14	1	25	18	Lower back	F3	-
133	6.00	19.68	2	70	360	Knee (bilateral)	F3/F4	-
134	3.80	11.24	2	72	516	Lower back	F4	-
135	4.36	13.71	2	76	84	Left shoulder	FC6	-
136	5.50	21.78	1	71	240	Lower back	F4	Yes
137	5.25	12.73	2	60	240	Lower back	F4	Yes
138	0.00	8.18	2	73	0	–	FC5	No
139	6.40	14.78	2	59	228	Lower back	F4	Yes
140	8.00	33.82	1	39	24	Lower back	F4	Yes
141	4.36	19.70	2	57	360	Lower back	F4	-
142	0.00	7.60	2	62	456	Lower back	F4	Yes
143	5.00	23,49	1	68	192	Lower back	F4	Yes
144	3.75	14.95	1	74	300	Lower back	F3	Yes
145	7.25	18.60	2	67	648	Neck–shoulder	FC6	Yes
146	5.36	9.15	1	73	360	Neck–shoulder	FC5	Yes
147	2.31	12.82	1	70	360	Lower back	F4	Yes
148	1.91	15.49	1	70	156	Left hip	F4	Yes
149	2.27	17.59	1	48	96	Lower back	F3	Yes
150	4.27	12.48	2	64	300	Neck–shoulder	FC6	-
151	4.45	15.86	1	64	180	Neck–shoulder	FC5	-
152	2.64	12.83	1	44	84	Neck	T7	Yes
153	1.36	11.72	2	70	144	Neck–shoulder	FC5	Yes
154	7.70	16.26	1	67	12	Neck–shoulder	FC6	–
155	4.00	15.08	1	61	360	Lower back	F4	–
